# Single-exposure X-ray phase imaging microscopy with a grating interferometer

**DOI:** 10.1107/S160057752200193X

**Published:** 2022-03-15

**Authors:** Andreas Wolf, Bernhard Akstaller, Silvia Cipiccia, Silja Flenner, Johannes Hagemann, Veronika Ludwig, Pascal Meyer, Andreas Schropp, Max Schuster, Maria Seifert, Mareike Weule, Thilo Michel, Gisela Anton, Stefan Funk

**Affiliations:** a Erlangen Centre for Astroparticle Physics (ECAP), Friedrich-Alexander-Universität Erlangen-Nürnberg, Erwin-Rommel-Strasse 1, D-91058 Erlangen, Germany; b Diamond Light Source, Harwell Science and Innovation Campus, Oxfordshire OX11 ODE, United Kingdom; c Department of Medical Physics and Biomedical Engineering, University College London, London WC1E 6BT, United Kingdom; d Helmholtz-Zentrum Hereon, Max-Planck-Strasse 1, D-21502 Geesthacht, Germany; e Center for X-ray and Nano Science CXNS, Deutsches Elektronen-Synchrotron DESY, Notkestrasse 85, D-22607 Hamburg, Germany; f Helmholtz Imaging Platform, Deutsches Elektronen-Synchrotron DESY, Notkestrasse 85, D-22607 Hamburg, Germany; g Institute of Microstructure Technology, Karlsruhe Institute of Technology, Hermann-von-Helmholtz-Platz 1, D-76344 Eggenstein-Leopoldshafen, Germany

**Keywords:** phase contrast X-ray imaging, X-ray microscopy, grating interferometry, phase retrieval

## Abstract

The advent of hard X-ray free-electron lasers enables nanoscopic X-ray imaging with sub-picosecond temporal resolution. X-ray grating interferometry offers a phase-sensitive full-field imaging technique where the phase retrieval can be carried out from a single exposure alone. Thus, the method is attractive for imaging applications at X-ray free-electron lasers where intrinsic pulse-to-pulse fluctuations pose a major challenge. In this work, the single-exposure phase imaging capabilities of grating interferometry are characterized by an implementation at the I13-1 beamline of Diamond Light Source (Oxfordshire, UK). For comparison purposes, propagation-based phase contrast imaging was also performed at the same instrument. The characterization is carried out in terms of the quantitativeness and the contrast-to-noise ratio of the phase reconstructions as well as via the achievable spatial resolution. By using a statistical image reconstruction scheme, previous limitations of grating interferometry regarding the spatial resolution can be mitigated as well as the experimental applicability of the technique.

## Abstract 2

This work presents and extends the single-exposure phase imaging capabilities of X-ray grating interferometry as demonstrated by an implementation at beamline I13-1 of the Diamond Light Source. The results are especially relevant for future single-pulse imaging experiments at X-ray free-electron lasers.

## 1. Introduction

Owing to its penetration power and its small wavelengths, hard X-ray imaging is particularly suited for nondestructive and quantitative probing of matter down to the nanometre scale, where it can give access to projective or volumetric structural information by two- or three-dimensional imaging, respectively. Consequently, much effort has been and still is put into improving the spatial resolution of X-ray imaging systems (Sakdinawat & Attwood, 2010 ▸; Momose, 2017 ▸). A fully quantitative understanding of samples, from the fields of biology or material sciences for instance, however, does not only require probing the structure of the respective specimens but also the study of the relevant dynamics in their governing processes. At synchrotron X-ray probes, time-resolved measurements have been carried out by either stroboscopic schemes or by high-frame-rate exposures with X-ray pulse lengths on the order of 100 ns from bunch trains down to around 100 ps from single pulses (Fezzaa & Wang, 2008 ▸; Rack *et al.*, 2014 ▸; Olbinado *et al.*, 2017 ▸; Parab *et al.*, 2018 ▸). The flux provided by individual synchrotron bunches is, however, not sufficient for certain applications, especially when the length scales of interest require nano-focusing of the X-ray beam (Hagemann *et al.*, 2021 ▸). Here, the advent of hard X-ray free-electron laser (XFEL) sources (Emma *et al.*, 2010 ▸; Ishikawa *et al.*, 2012 ▸; Tschentscher *et al.*, 2017 ▸) presents further advances, as imaging with sub-picosecond pulses prevents even the smallest degradations due to sample movements and because it allows to bypass existing limits imposed by radiation damage (Neutze *et al.*, 2000 ▸).

In the hard X-ray regime, the real part of the refractive index, which governs the phase shift due to the sample, can be orders of magnitude larger than the imaginary part, which sets the absorption (Fitzgerald, 2000 ▸). This is of special relevance for the study of materials with low atomic numbers *Z* as well as for micro- and nanoscopic imaging where specimens show little or no attenuation contrast (Withers, 2007 ▸). Many of the realized phase-sensitive imaging techniques, *e.g.* ptychography (Rodenburg *et al.*, 2007 ▸) or analyzer-based imaging (Ingal & Beliaevskaya, 1995 ▸; Davis *et al.*, 1995 ▸), cannot be reconciled with single-exposure acquisition schemes. The referenced single-pulse experiments at synchrotron sources either employed simple X-ray radiography or propagation-based phase contrast imaging (PBPCI) (Snigirev *et al.*, 1995 ▸; Cloetens *et al.*, 1996 ▸, 1999 ▸). The latter approach has also found first applications at XFELs (Schropp *et al.*, 2015 ▸; Vagovič *et al.*, 2019 ▸; Hagemann *et al.*, 2021 ▸).

Grating or Talbot interferometry (David *et al.*, 2002 ▸; Momose *et al.*, 2003 ▸) represents another phase-sensitive imaging technique. While the predominantly used variant of the grating-based method is based on the phase stepping or fringe scanning technique (Weitkamp *et al.*, 2005 ▸), and thus on the acquisition of multiple frames, it is also possible to extract phase contrast images from a single exposure through the application of the Fourier transform method from Takeda *et al.* (1982 ▸), either directly to the interference pattern (Bennett *et al.*, 2010 ▸) or to a moiré pattern created thereout (Momose *et al.*, 2009 ▸). A downside of this single-exposure approach is the requirement of effectively band-limited signal components in order to separate phase and amplitude information, which limits the achievable spatial resolution to half-periods equal to the demagnified fringe period (Takeda *et al.*, 1982 ▸; Momose *et al.*, 2009 ▸). At XFELs, grating interferometry has therefore found its main application in the field of wavefront sensing (Rutishauser *et al.*, 2012 ▸; Nagler *et al.*, 2017 ▸; Seaberg *et al.*, 2019 ▸; Makita *et al.*, 2020 ▸). Here, assuming sufficiently slow spatial variations is more appropriate regarding the illuminating wavefront than for microscopic samples. Furthermore, grating interferometers can be incorporated into full-field X-ray microscopes (Takeda *et al.*, 2008 ▸; Yashiro *et al.*, 2009 ▸; Berujon *et al.*, 2012 ▸). For interferometers sensitive to the phase gradient, there is a trade-off between the achievable angular sensitivity and the magnification of the microscope. Yashiro *et al.* (2009 ▸) remedied this issue by designing the interferometer around a single grating and a highly magnified Talbot self-image. With such a layout, one can obtain phase difference instead of differential phase images. The reconstruction techniques proposed for phase imaging in this regime (Yashiro *et al.*, 2009 ▸, 2010 ▸) are however subject to some restrictions, for instance the requirement of a quasi uniform amplitude of the investigated wavefield.

In order to broaden the applicability of grating interferometry in the phase difference regime, we recently proposed a statistical image reconstruction approach based on a maximum-likelihood estimation and validated the method on the basis of simulated data (Wolf *et al.*, 2020 ▸). The scheme is based on a full forward model of the image formation process due to the interferometer and thus avoids the above-mentioned constraints. Furthermore, our ansatz does not rely on the extraction of single Fourier order terms from an interferogram. Hence, the achievable spatial resolution is not limited a priori. In this work, we demonstrate the single-exposure imaging capabilities of grating interferometry with our novel algorithmic approach on the basis of experimental data obtained at a synchrotron radiation X-ray source. We study the achievable image quality in different configurations of the X-ray microscope as well as for several layouts of the interferometer. Comparative measurements with propagation-based phase contrast imaging were realized at the same instrument.

The paper is structured as follows. Section 2 describes the experimental setup at beamline I13-1 of the Diamond Light Source. Section 3 details the course of data evaluation from the data acquisition to data pre-processing to the final reconstruction of phase images for both grating interferometry and propagation-based imaging. In Section 4, we present the results of the imaging experiments. We close the manuscript with a summary and outlook in Section 5.

## 2. Experimental implementation at I13-1

**Figure 1:**
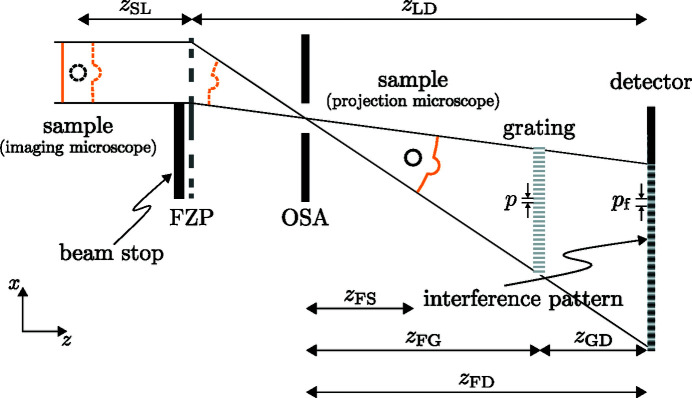
Schematic of the setup. The X-rays enter from the left and are focused by a partially covered Fresnel zone plate (FZP). An order sorting aperture (OSA) is positioned in the back focal plane of the FZP in order to minimize the impact of higher diffraction orders from the FZP. The grating with period *p* is placed in the divergent beam such that a magnified self-image with fringe period *p*_f_ occurs in the detection plane. The sample can be either placed downstream of the focus, corresponding to a projection microscope and suggested by the solid circle, or in a conjugate plane upstream of the FZP as implied by the dashed circle, resulting in an imaging microscope. Specific distances of the setup, as they are introduced in the main text, are indicated.

The experiment was carried out at the coherence branch of beamline I13 of the Diamond Light Source (Rau, 2017 ▸). The X-ray beam, generated with an undulator placed more than 200 m away from the experimental hutch, was monochromated to an X-ray energy of 10 keV using a Si-111 double-crystal monochromator. The experimental setup is shown in Fig. 1 ▸. Following Yashiro *et al.* (2010 ▸), we differ between an imaging and a projection microscope. In the former case, the sample and the X-ray detector are placed in conjugate planes of the focusing element. Thus, the distances *z*_SL_ between the sample and the focusing element and *z*_LD_ between the focusing element and the detector meet the classical lens law 1/*z*_SL_ + 1/*z*_LD_ = 1/*f* from geometrical optics (Goodman, 2005 ▸) with *f* the focal length. Such a configuration leads to the formation of a real sample image in the detection plane. For a projection microscope, on the other hand, the sample is positioned in the divergent beam downstream of the focusing element. In this case, Fresnel diffraction images of the sample will be measured in the detection plane. Both variants are indicated in Fig. 1 ▸.

During our experiment, the X-rays were focused by a Fresnel zone plate (FZP) with a diameter of 70 µm and an outer zone width of 150 nm, resulting in a focal length *f* = 8.5 cm at 10 keV. The choice of this rather small FZP was necessary since the sample magnification of the imaging microscope scales inversely with *f* and hence inversely with the FZP diameter for a fixed overall distance *z*_LD_. Note that for the projection microscope, on the other hand, the achievable sample magnification is mainly limited by the numerical aperture of the FZP which only depends on the wavelength and the outermost zone width. A knife edge was placed upstream of the FZP as a beam stop covering slightly more than half of its area and thus effectively blocking off the non-diffracted beam. In order to further minimize the influence of higher diffraction orders from the FZP, an order sorting aperture (OSA) with a diameter of 10 µm was positioned in its back focal plane.

The grating for the X-ray interferometer was manufactured at the Institute of Microstructure Techology of the Karlsruhe Institute of Technology. It was produced as a binary line grating using the deep X-ray lithography process (Reznikova *et al.*, 2008 ▸; Meyer & Schulz, 2015 ▸). The resist (MRX-10, micro resist technology GmbH) is an epoxy-based one similar to SU-8, while the substrate is a 500 µm-thick polyimide wafer. The grating has a nominal period *p* = 10 µm and a nominal resist height of 25 µm approximately resulting in a −π phase shift at the employed X-ray energy. The grating was mounted on a moveable stage in the divergent beam generated by the FZP. According to the fractional Talbot effect for spherical wave illumination, self-images of the grating are formed upon placing the grating at a distance 

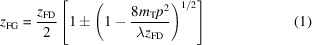

downstream of the focus (Yashiro *et al.*, 2009 ▸). Here, λ denotes the X-ray wavelength, *m*_T_ the fractional Talbot order, and *z*_FD_ the distance between the focal plane and the detector. For the solution with the minus sign in equation (1), the available range of the grating stage enabled the realization of Talbot distances between *m*_T_ = 5/16 and *m*_T_ = 11/16. The resulting grating magnifications *M*_GD_ = *z*_FD_/*z*_FG_ were large enough in order to resolve the respective fringe pattern directly.

For the X-ray detection, an sCMOS camera (Hamamatsu C12849-111U) with 2048 × 2048 pixels and a pixel size of 6.5 µm was positioned at a distance *z*_LD_ ≃ 14.5 m downstream of the focusing optics. The camera uses a 10 µm-thick Gadox scintillator that is coupled via fibre optics to the sCMOS chip. In order to reduce scattering and absorption in air, a flight tube filled with helium gas was set up between the grating and the detector. The samples were either placed in the conjugate plane, *i.e.* around 8.6 cm upstream of the FZP for the imaging microscope, or in the divergent beam at a distance *z*_FS_ = *f* resulting in the same sample magnification for the projection microscope. As for the comparative measurements with propagation-based phase contrast, the samples were kept in the downstream position from the projection microscope and the grating was removed from the beam path. A detailed characterization of similar PBPCI setups based on FZPs was recently presented by Flenner *et al.* (2020 ▸). For a precise alignment, the sample, the grating, and all the optical elements were mounted on stick-slip piezo stages (SmarAct).

Regarding the alignment of the interferometer, the grating was first placed at the distance *z*_FG_ expected from equation (1) for the respective Talbot order. Subsequently, the grating was scanned in a region around this position and an image was acquired for each step. This procedure does not only allow optimizing the grating position to obtain both a high fringe contrast and uniformity of the interference pattern across the field of view but it also enables an accurate calibration of the imaging geometry. The latter point is crucial, as accurate propagation distances are required for the image reconstruction, *cf*. Section 3.

Following a similar calibration approach from Bartels (2013 ▸), the two distances *z*_FD_ and *z*_FG_ are assumed to be unknown, whereas the relative displacement Δ*z* of the grating can be obtained from the motor positions of the moveable stage. The inverse grating magnification can then be written as a linear function 

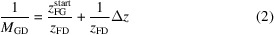

of the displacement Δ*z* with 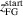 the position of the grating at the beginning of the grating scan. Experimentally, the grating magnification can be determined from the known grating period *p* and the fringe frequency ν_f_ corresponding to the dominant *q*th Fourier order of the measured interference pattern, *i.e.*


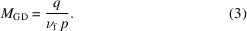

The spatial frequency ν_f_ is obtained from the highest peak of the Fourier transformed intensity apart from the DC component. An uncertainty corresponding to half the sampling interval in Fourier space is assumed for ν_f_ and Gaussian error propagation is applied to obtain the error of the inverse magnification.

**Figure 2:**
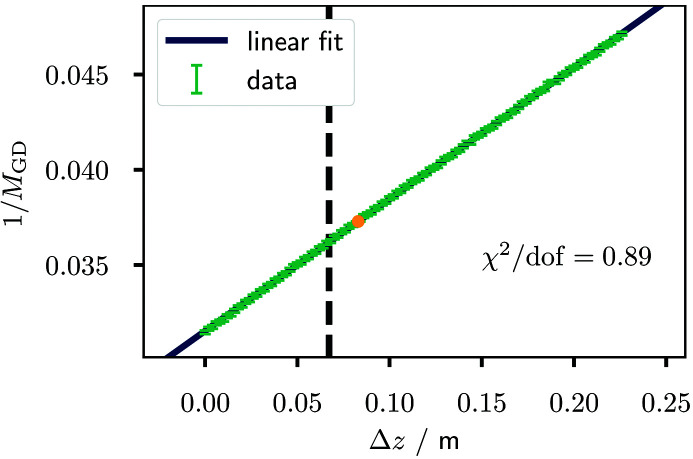
Linear fit to the inverse grating magnification for the grating scan around the fractional Talbot order *m*_T_ = 5/16 plotted against the displacement Δ*z* as introduced in equation (2). The focus-to-detector and the focus-to-grating distance determined from the fit can be found in Table 1 ▸, together with the results for the other three realized Talbot orders. The theoretically expected position for a −π-shifting grating is indicated by the vertical dashed line. The orange dot marks the grating position used in the subsequent imaging experiments.

An exemplary linear fit to the inverse magnification values is shown in Fig. 2 ▸ for the grating scan around the fractional Talbot order *m*_T_ = 5/16. Overall, the data are in good agreement with the expected linear relationship. From equation (2), the unknown distances *z*_FD_ and 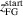 can be calculated using the slope and the *y*-intercept obtained from the fit. The results are listed in Table 1 ▸. The respective uncertainties are obtained by Gaussian error propagation starting from the covariance matrix of the fit parameters. It is worth mentioning that the grating positions chosen upon a visual inspection of the grating scan data partly differ from the theoretically ideal distances for a −π-shifting grating. Exemplarily, such a deviation is indicated in Fig. 2 ▸ as well. They are suspected to result from variations in the grating height.

## 3. Data acquisition and evaluation

During the imaging experiments, a series of *N*_f_ = 10 frames was recorded for each sample and each reference measurement. The idea behind this scheme was to acquire a sufficiently high signal-to-noise ratio while also being able to detect beam fluctuations or possible vibrations of optical elements over short time scales. In practice, only small variations were discernible over the duration of an exposure series. On the other hand, long-term changes regarding the position of the beam, the photon flux, and clipping of the beam by optical elements were observed during the beam time. In part, these effects could be compensated by progressively realigning the optical elements, *i.e.* the FZP, the OSA and the beamstop. Regarding the flux, the exposure time of the individual frames was adjusted to warrant at least a rough comparability between different measurements, *e.g.* imaging of the same sample with the grating aligned in various Talbot orders. The overall decline in flux was later found to be caused by a broken chiller of the monochromator which led to overheating. For the results presented hereinafter, the exposure time was varied within 2 s and 3 s per frame.

**Figure 3:**
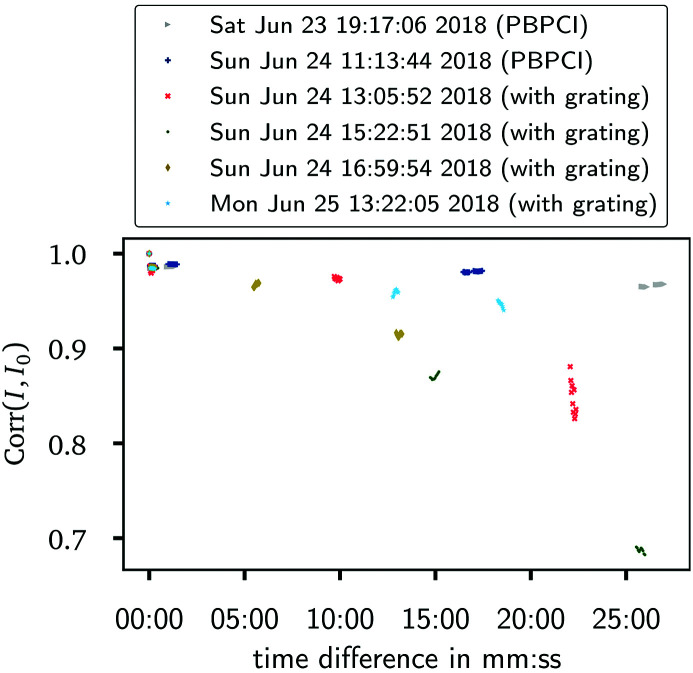
Similarity between different reference frames as assessed by temporal changes in their correlation coefficient. A new starting frame was defined whenever the setup was changed, leading to the depicted grouping. For each group the time stamp of the first acquisition is specified in the legend.

The inconstancy of the illumination is illustrated in Fig. 3 ▸. There, the correlation coefficient 

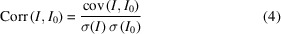

between two different reference frames is plotted over the time difference between their respective acquisitions. Exposure series falling in between realignments of the optical elements or the grating are grouped together. In equation (4), *I*_0_ then denotes the first reference frame of each such group, cov the covariance of two exposures and σ the standard deviation over a single acquisition. As can be seen in Fig. 3 ▸, there is typically a larger decrease in correlation in between two exposure series as compared with a single series itself. In comparison with propagation-based measurements, a faster decrease in correlation was observed for the measurements with the grating interferometer. On the one hand, this can be attributed to the above-mentioned problems with the chiller starting during the time span of the grating measurements. On the other hand, the chronologically last group of exposure series comprises measurements with a newly installed chiller and still manifests a worse stability in relation to the exposures without the grating in the beam. Whether this is due to a higher sensitivity of the grating-based setup to the remaining changes in the X-ray beam or due to fluctuations regarding the positioning of the grating itself cannot be definitively answered here.

Exposure series including a sample in the beam temporally fall in between the reference series shown in Fig. 3 ▸. Consequently, larger changes in the illumination between sample and reference measurements have to be expected here as well. In view of this, the smaller changes within a single series can be ignored and the *N*_f_ frames of each series were averaged prior to further processing. The choice of this procedure is further supported by the visual inspection of reconstructions from maximally correlated single sample and reference frames not revealing any improvements. During the subsequent processing, an appropriate dark frame, recorded with closed beam shutter, was substracted from each averaged frame. As a last point prior to the actual reconstruction, the resulting pixel values were clipped to zero in order to avoid negative numbers of counts.

The phase retrieval for measurements with the grating interferometer was carried out using the statistical image reconstruction (SIR) method introduced by Wolf *et al.* (2020 ▸). There, the image formation process is modelled via a double sum over the grating diffraction orders. The resulting intensity is then given by 

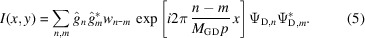

Here, 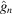 = 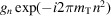 with *g*_*n*_ the *n*th Fourier coefficient belonging to the complex transmission function of the grating. Ψ_D,*n*_ = Ψ_D_(*x* − *nd*_s_, *y*) represents the wavefield that would arrive at the detector in the absence of the grating, but translated about *n* times the shear distance 

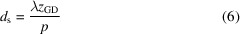

between adjacent diffraction orders. In comparison with the original validation study on idealized simulated data from Wolf *et al.* (2020 ▸), a Gaussian weighting term 

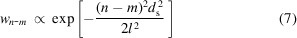

was included in the forward model in order to reproduce reductions to the fringe contrast due to partial spatial coherence and the point spread function of the detector. Together with the position of the grating, the width *l* required in that term was fitted in advance for each reconstruction, when possible to a subset of the intensity data containing no sample. The remaining parameters of the forward model are either assumed to be known, *i.e.* the X-ray wavelength and the grating period, or they can be obtained with the aid of the calibrated distances from Table 1 ▸. The actual image reconstruction then involves the maximization of the likelihood with respect to the wavefield Ψ_D_ given the respective measured intensities *I*_meas_. Thereby, Ψ_D_ is expressed through its amplitude and phase values *A* and ϕ over the pixel matrix of the detector. Numerically, the optimization is handled through the minimization of the corresponding negative log-likelihood function. Due to the characteristics of scintillation-based detectors, *cf*. the discussion by Stampanoni *et al.* (2002 ▸), Gaussian statistics were assumed instead of the Poisson likeli­hood from Wolf *et al.* (2020 ▸). In order to impose a certain degree of smoothness on the final results, anisotropic ε-smoothed total variation norms (Acar & Vogel, 1994 ▸), namely 

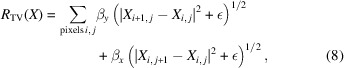

for both the amplitude, *i.e.* *X* = *A*, and the phase, *i.e.* *X* = ϕ, were added to the negative log-likelihood function as regularization terms. The total cost function then reads 

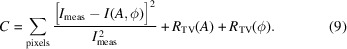

Here, *I*(*A*, ϕ) is the forward model from equation (5) which is now seen as a function of the amplitude *A* and the phase ϕ of the wavefield Ψ_D_ = 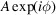 for each pixel rather than as a function of the pixel coordinates. The parameters of the TV regularization from equation (8) were set to β_*x*_ = 0.5, β_*y*_ = 0.05 and ε = 10^−4^ for both *A* and ϕ. The anisotropy resulting from different values for β_*x*_ and β_*y*_ was opted for due to the directionality introduced by the employed line grating (Wolf *et al.*, 2020 ▸). Grating diffraction orders up to |*n*| = 7 were used in the forward model and the minimization of the cost function was terminated after reaching the threshold 10^−3^ for the norm of the gradient or the maximum of 1000 iterations of the L-BFGS-B algorithm (Zhu *et al.*, 1997 ▸).

**Figure 4:**
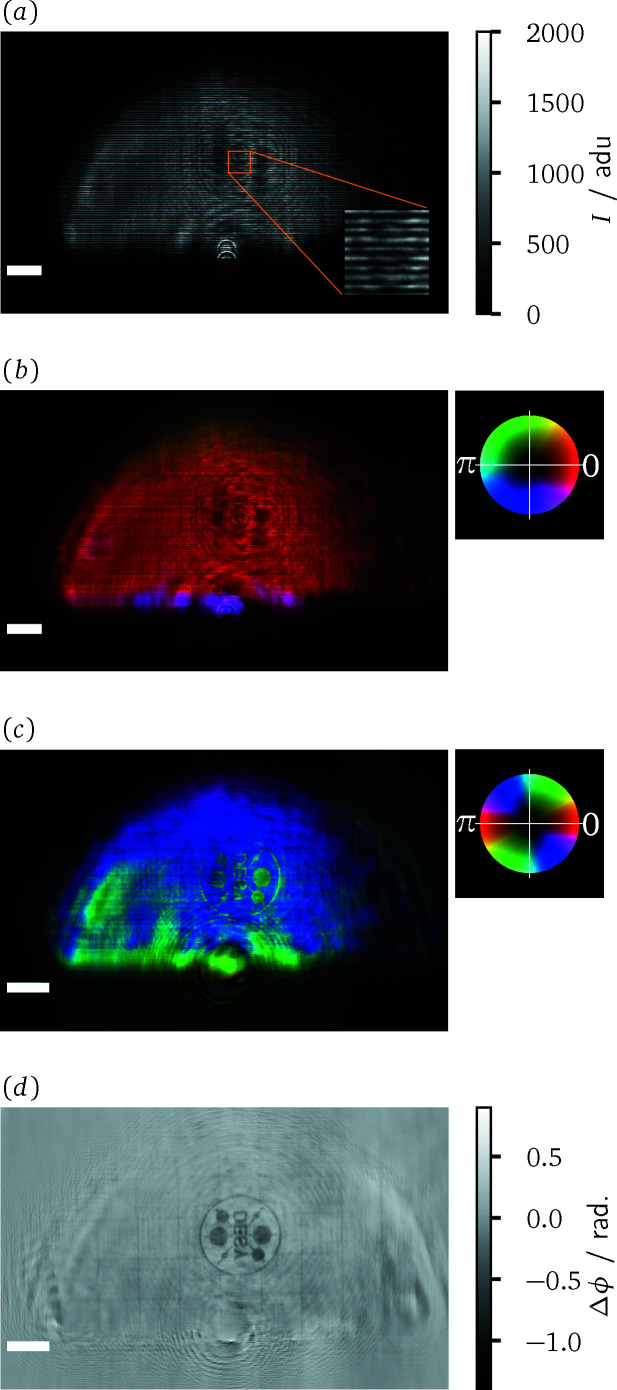
Overview of the data evaluation for grating interferometry. (*a*) Dark frame corrected raw data for a sample measurement in analog digital units (adu) of the sCMOS camera. The inset highlights the interference pattern over a region of 100 × 100 pixels. (*b*) Complex wavefield reconstructed from this single-exposure data via the SIR method. The amplitude is mapped to brightness and the phase to hue, as indicated by the adjacent colour wheel. (*c*) Wavefield after backpropagation to the sample plane. (*d*) Phase shift of the sample after reference correction. The scale bar indicates 1 mm length in panels (*a*) and (*b*) and 10 µm in (*c*) and (*d*).

An exemplary course of the data evaluation, from the measured intensities to the reconstructed phase image of the sample, is illustrated in Fig. 4 ▸ for a measurement with the projection microscope. Panel (*a*) shows the averaged and dark frame corrected data for a sample measurement of a DESY-logo test pattern. The test pattern is made out of a 500 nm-thick gold layer on a 200 nm silicon nitride membrane and has a diameter of 20 µm. The inset in Fig. 4 ▸(*a*) zooms in on the interference pattern over a region of 100 × 100 pixels. It showcases how the fringe pattern of the grating is deformed due to the presence of the sample. The amplitude and phase maps resulting from the SIR method are combined to form a complex-valued wavefield. Panel (*b*) shows this reconstructed wavefield Ψ_D_ in the plane of the detector. There, the phase is encoded in the hue and the amplitude in the brightness of the image, as can be inferred from the adjacent colour wheel. Noticeably, some stripe artefacts appear in the reconstructed amplitude. They are presumed to originate from intensity variations across the fringes of the interference pattern. A uniform grating, as assumed in the forward model of the SIR method, cannot reproduce these variations without proportional differences in the amplitude of the underlying wavefield. For measurements with a projection microscope, the wavefield from panel (*b*) needs to be propagated back into the sample plane. The result of this operation is depicted in panel (*c*). When employing an imaging microscope, Ψ_D_ corresponds to a magnified and mirrored version of the exit surface wave of the sample. Hence, the backpropagation step can be skipped or it can be used to correct potential defocusing of the setup. Modifications of the phase by the phase shift of the sample are visible in (*c*), even though they are partly obstructed by features of the illumination. Note that, in comparison with panel (*b*), the phase encoding via the hue was adjusted in order to accentuate the sample features. The complex transmission function of the sample can be recovered by forming the quotient of the wavefields from panel (*c*) and the corresponding result obtained from a reference measurement. The magnitude of the quotient yields the amplitude transmission and the argument the phase shift of the sample. The latter is displayed in panel (*d*).

In order to obtain valid results from the last step, the temporal stability of the illumination between both measurements is crucial. For the present case, as expected from the earlier correlation analysis, the phase shift image features multiple signal components that cannot be attributed to the test pattern. Evidently, larger reconstruction errors have to be expected for the regions at and beyond the boundary of the X-ray beam. In these regions, the amplitude rapidly falls off such that they are most susceptible to changes in the positioning of the beam. Additionally, points with low amplitude cannot contribute significantly to the intensity images. Conversely, the phase values at these points are mainly set by the regularization rather than the measured data. Furthermore, one can observe a faint low-frequency modulation of the phase values within the extent of the beam. Besides such effects due to the instability of the illumination, hints of the twin-image from in-line holography (Nugent, 1990 ▸) can also be perceived in the area surrounding the test pattern. This may indicate that the SIR algorithm did not recover the phases of Ψ_D_ fully correctly, but instead stagnated in a local minimum. Despite these aspects, the test pattern is still clearly visible. Furthermore, the phase image reveals a grid-like substructure of the test pattern wafer.

The phase retrieval for the comparative PBPCI measurements is performed by employing the iterative algorithm of relaxed averaged alternating reflections (RAAR) (Luke, 2005 ▸). As most iterative phase retrieval techniques, this scheme is based upon two constraints: the measurement constraint which forces the exit surface wave Ψ of the sample to be consistent with the measured intensity image and a sample domain constraint which imposes a priori knowledge about the imaged object. In RAAR, the current iterate is not directly projected onto the solution sets of the two constraints. Instead of projectors *P*_M/S_, the wavefield is mainly acted on by the associated reflectors 

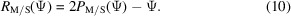

Overall, the new iterate of the wavefield from RAAR follows from the fixed point iteration 

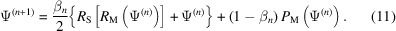

Here, the relaxation behaviour of the algorithm can be tuned by varying the parameter β_*n*_. Evidently, the new iterate will follow the measurement more closely for smaller values of β_*n*_. This is especially advantageous in the case of inconsistent problems, *i.e.* when the intersection of the solution sets for the two employed constraints is empty. The choice 0 < β_*n*_ < 1 then leads to a damping of the iterations and ensures the existence of fixed points. In this work, the dynamic strategy 

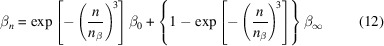

from Hagemann & Salditt (2017 ▸) with the parameters β_0_ = 0.99, β_∞_ = 0.75 and *n*_β_ = 150 is employed. The standard projector *P*_M_ for the projection on the measurements is used. It consists of Fresnel propagation into the detection plane, substitution of the amplitude by the square root of the measured intensity, and the backpropagation to the sample plane. For the sample domain constraint enforced by *P*_S_, we utilize a finite support generated by thresholding and morphological operations. Furthermore, *P*_S_ was chosen to impose a non-positive phase on one side as well as a non-negative amplitude absorption or alternatively a pure phase object on the other side.

When applied to PBPCI, RAAR is reliant on the empty beam division, where a hologram or a normalized intensity is generated by dividing sample and reference measurements, *i.e.* 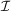 = *I*_obj_/*I*_ref_. This correction step is supposed to artifically generate conditions that are equivalent to the ideal illumination by a point source. The exit surface wave can then be identified with the complex transmission function of the sample, which is a necessary requirement for most sample domain constraints to be applicable. However, the empty beam division thereby also assumes that the effects of free space propagation can be separated for the sample and the illumination. This assertion was investigated in detail by Homann *et al.* (2015 ▸), with the result that the errors introduced by the approximation increase for larger source sizes, *i.e.* less smooth wavefronts, as well as for smaller relevant length scales in the sample.

**Figure 5:**
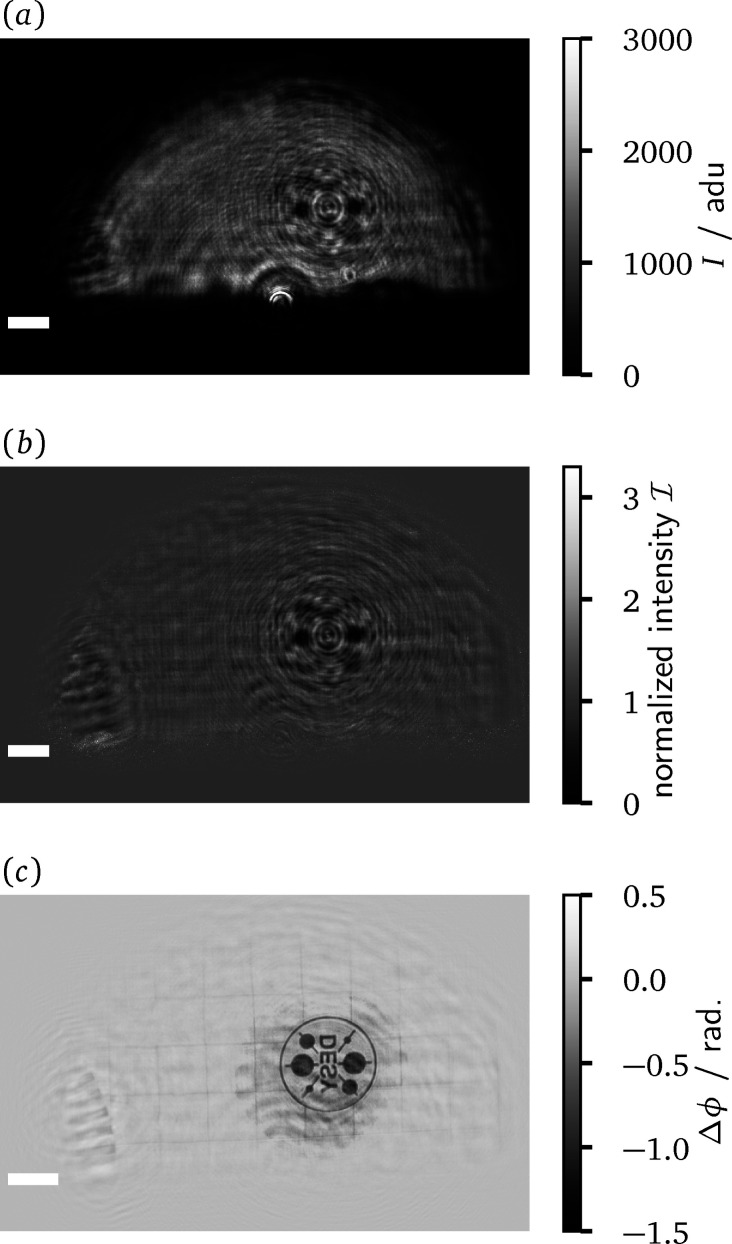
Overview of the data evaluation for PBPCI. (*a*) Dark frame corrected raw data for a sample measurement in analog digital units (adu) of the sCMOS camera. (*b*) Normalized intensity, obtained by the empty beam division. (*c*) Phase reconstruction obtained with the RAAR scheme from (*b*). The scale bar indicates 1 mm length in the detection plane for panels (*a*) and (*b*) and 10 µm in the sample plane for (*c*).

The data evaluation for PBPCI is illustrated in Fig. 5 ▸. Panel (*a*) shows a dark frame corrected sample exposure for the DESY-logo test pattern. The normalized intensity 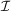 , which serves as an input for RAAR, is displayed in panel (*b*). In order to mimic the illumination with an undisturbed plane wave, the normalized intensity obtained from the quotient of (*a*) and a suitable reference frame was smoothly transitioned to a constant value of unity for the area outside the illuminated semicircle. The resulting phase reconstruction of the test pattern is visualized in Fig. 5 ▸(*c*). Similar to the grating-based reconstruction, the phase shift of the test pattern is correctly recovered. Within the support of the reconstruction, artefacts in the form of low-frequency phase modulations appear. They are again presumed to originate from beam fluctuations between the sample and reference measurements. Outside the support, these artefacts are suppressed. However, also sample features beyond the support region, like the grid-like structure of the wafer or the marginal area of a Siemens star in the lower left corner, are not correctly recovered. These components were not captured during the initial setup of the support. Here, dynamic support adaptions (Marchesini *et al.*, 2003 ▸; Hagemann *et al.*, 2021 ▸) starting from a less constrictive initial guess might offer room for improvements.

## 4. Imaging results

### 4.1. Imaging microscope with OSA

**Figure 6:**
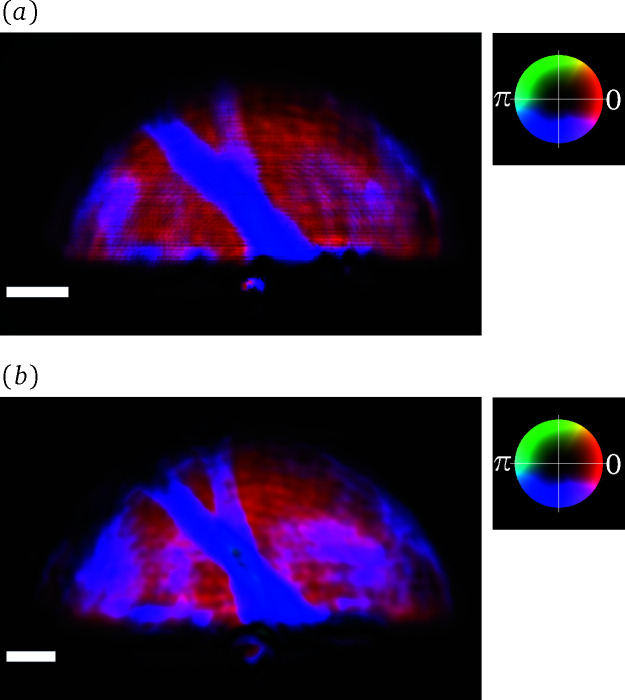
Grating-based imaging microscope with OSA. Panel (*a*) shows the retrieved wavefield in the sample plane of the imaging microscope with *m*_T_ = 11/16. The analogous result from the projection microscope is shown in (*b*). There, the wavefield has been low-pass filtered in Fourier space using the expected diameter of the OSA. The phase shift following from the non-filtered reconstruction is depicted in Fig. 7 ▸(*a*). For both panels, the scale bar indicates 10 µm length in the respective sample plane. Note that the sample magnification as well as the positioning of the carbon fibres within the field of view differ between both exposures.

**Figure 7:**
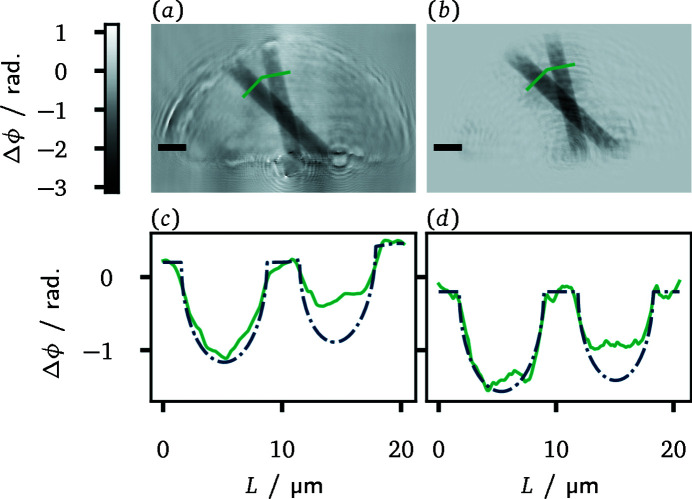
Phase reconstructions of the crossed carbon fibre sample via grating interferometry in a projection microscope (*a*) with *m*_T_ = 11/16 and via PBPCI (*b*). The scale bar indicates 10 µm length in both panels. Panels (*c*) and (*d*) show section profiles along the line segments indicated in (*a*) and (*b*), respectively (green curves). The expected profile for carbon fibres with a diameter of 7.2 µm and 6.4 µm is shown for comparison (blue dash-dotted curve). Note, that the expected profile plot was adjusted to the baseline of the reconstructions.

Regarding the imaging microscope with grating interferometry, an inferior spatial resolution in comparison with the projection microscope became apparent. This observation is illustrated in Fig. 6 ▸ taking the example of two crossed carbon fibres as sample. In panel (*a*) the reconstructed wavefield in the sample plane of the imaging microscope is displayed. While both fibres as well as their overlap area can be identified, the sample features appear blurred out. In contrast, the reconstruction of the same sample from grating interferometry in the projection microscope, *cf*. Fig. 7 ▸(*a*), is significantly sharper. A possible explanation for the degraded resolution is provided by the OSA. The latter acts as a low-pass filter in the focal plane of the FZP. In the paraxial approximation, an aperture with diameter *d*_OSA_ will cut off spatial frequencies above ν_cutoff_ = *d*_OSA_/2λ*f* (Goodman, 2005 ▸). For the setup at hand, the cutoff amounts to ν_cutoff_ = 1/2.1 µm^−1^ which is far more restrictive than other limitations by the detector or the numerical aperture of the FZP. Regarding Talbot interferometry with an imaging microscope, the only other work explicitly mentioning the use of an OSA that is known to the authors is from Berujon *et al.* (2012 ▸). There, the inverse cutoff frequency is only four to five times larger than the demagnified pixel size, compared with a factor of around 40 in this work. Assuming that the point spread function of the detection system exceeds the pixel size by a similar amount, as for instance in Desjardins *et al.* (2014 ▸), detrimental effects due to the OSA might not be as apparent. In order to further substantiate the assumption that the limited resolution is caused by the OSA, a corresponding reconstruction using the projection microscope was low-pass filtered according to the calculated cutoff frequency. The result is shown in Fig. 6 ▸(*b*). The degree of blurriness regarding the sample features is comparable for both images. When comparing them, note that both the alignment of the two carbon fibres within the field of view as well as their magnification differ between the two employed imaging geometries. Regarding the sample magnification in the projection microscope, the condition *z*_FS_ = *f* for the distance between the focal plane and the sample was not met, resulting in a smaller magnification of the fibres.

### 4.2. Quantitative contrast

The carbon fibre sample presented above is suited to characterize the quantitative accuracy of the imaging methods. Reconstructions of the crossed fibres with the grating-based projection microscope as well as from propagation-based phase contrast measurements are displayed in Fig. 7 ▸. The refractive index decrement for carbon fibres with a mass density of 1.8 g cm^−3^ at 10 keV is δ = 3.74 × 10^−6^ (Henke *et al.*, 1993 ▸). For the employed carbon fibres with diameters between 6 µm and 8 µm, this results in a maximal expected phase shift between Δϕ = −1.14 and Δϕ = −1.52. Taking offsets in the baseline of the reconstructed phase into account, these expectations fit closely to the reconstructed values.

The quantitative character of the phase reconstructions is further illustrated by section profiles shown in Figs. 7 ▸(*c*) and 7(*d*). There, the reconstructed phase shift values are plotted over the length of the line segments marked in panels (*a*) and (*b*). The section profiles are approximately consistent with fibre diameters of 7.2 µm and 6.4 µm, respectively, albeit the reconstructed phase shift of the smaller fibre lies beneath the expectation for both methods. Note that offsets as well as gradients regarding the baseline of the reconstruction in the vicinity of the fibres were incorporated in the expected line profiles. For grating interferometry, the underestimation of the phase shift can be due to the orientation of the fibres. The smaller fibre is aligned more in the direction perpendicular to the grating bars. Since the interferometer is only sensitive to phase differences along that direction, phase information obtained from the end points of the fibre becomes more pivotal for the overall reconstruction. The end points, however, lie outside the field of view. With respect to the larger fibre, propagation-based imaging achieves a sharper reconstruction of the fibre sidewalls. The central region of the fibre, on the other hand, is reproduced more reliably by the grating-based method.

### 4.3. Imaging at different Talbot orders

Hereinafter, the achievable image quality with the projection microscope is to be compared for the different realized Talbot orders *m*_T_ of the grating interferometer. A change of the Talbot order alters three important parameters of the setup that can potentially impact the image quality of the reconstructions: the shear distance *d*_s_, the fringe period *p*_f_ of the interference pattern, and its visibility *V*. For the present setup, the grating was always mounted in a position corresponding to a solution of equation (1) with the minus sign. Thus, moving towards higher Talbot orders, *i.e.* longer effective propagation distances, implies larger focus to grating distances *z*_FG_. Consequently, higher Talbot orders go along with shorter shear distances and smaller fringe periods. The dependency of the visibility cannot be predicted as generally, as there are two effects working in opposite directions. In the case that the spatial coherence limits the visibility, improvements can be expected for higher Talbot orders. If, on the other hand, the resolution of the detector is the limiting factor, higher visibilities should be obtained for larger fringe periods and hence for smaller values of *m*_T_. The interplay of both factors is similar to that discussed for the propagation-based phase contrast in Pogany *et al.* (1997 ▸). In the case of the present setup, the general trend showed an overall decrease in visibility for higher Talbot orders, thus indicating that the detector represents the limiting factor. The values of the mentioned parameters for all setup configurations can be found in Table 2 ▸.

The accuracy of the reconstructions is quantified using the contrast-to-noise ratio 

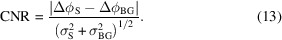

**Figure 8:**
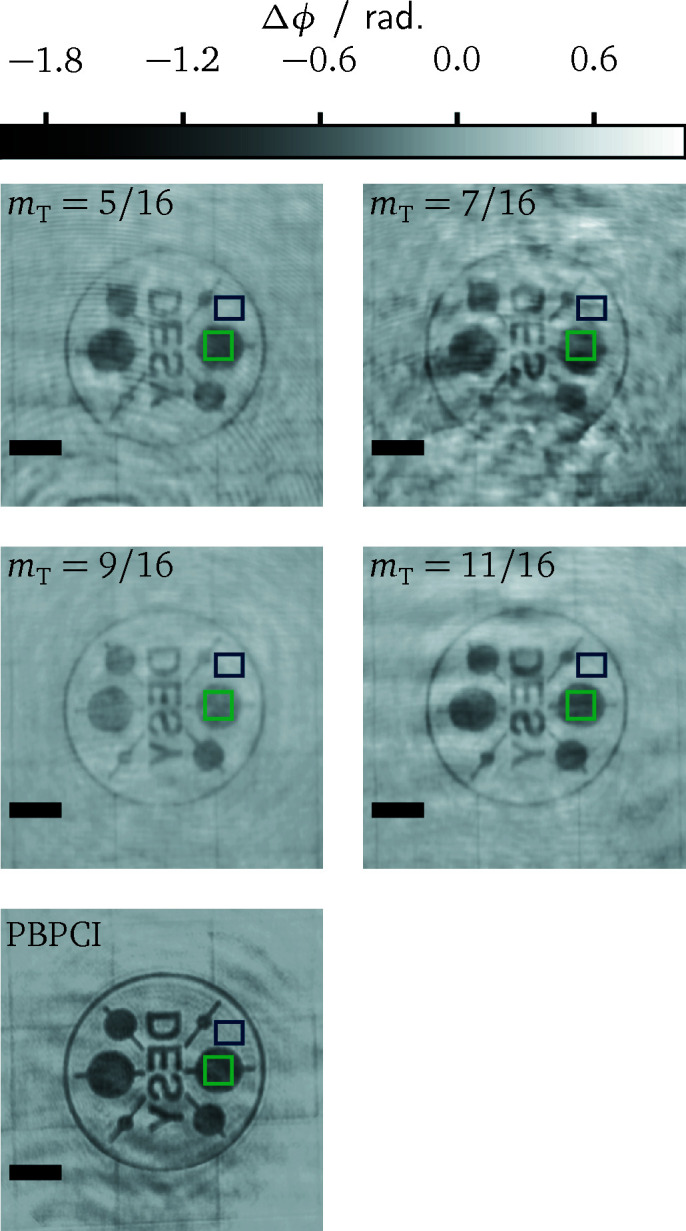
Phase reconstructions of the DESY-logo for different Talbot orders and for PBPCI. The regions used for the CNR evaluation are marked by the green and blue squares, respectively. The scale bar indicates 5 µm length in all panels.

Here, Δϕ_S_ and Δϕ_BG_ denote the average pixel values of the reconstructed phase image in a sample and a background region, respectively, 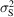 and 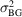 the corresponding variances. The test pattern with the DESY-logo, as shown in Figs. 4 ▸ and 5 ▸, represents a suitable sample for the CNR evaluation. The reconstructions from the different setup configurations are displayed in Fig. 8 ▸. There, the selected square regions of interest for the determination of the CNR values are marked in green and blue in all panels. One of the larger disks of the logo was chosen as sample region, since a constant phase shift can be expected there. The resulting CNR values are listed in Table 2 ▸. The highest values around CNR ≃ 8 are obtained for *m*_T_ = 5/16 and *m*_T_ = 11/16. For these two configurations, the achieved noise level lies slightly below the one of the purely propagation-based method. The CNR values for *m*_T_ = 7/16 and *m*_T_ = 9/16 are significantly worse. The phase reconstruction for *m*_T_ = 7/16 is spoiled by cloud-like features causing a severe increase of the variances 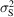 and 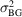 . Similar artefacts were observed for all acquisitions with this configuration. Whether they represent actual parts of the wavefield or whether they are artificially introduced by the reconstruction method, for instance through a stagnation of the likelihood maximization, could not be finally decided on. The case with *m*_T_ = 9/16, on the other hand, featured the smallest phase variances overall. Here, the contrast of the reconstruction is too small and responsible for the worse CNR.

From the experimental results, no clear correlation between the CNR values with either the shear distance or the visibility was found. In principle, as has been worked out previously by Weber *et al.* (2011 ▸) for example, an increase in either of the two quantities could improve the signal-to-noise ratio, at least for differential phase or phase difference images obtained with standard reconstruction approaches, where single Fourier orders of the interference pattern have to be extracted. A higher visibility should lead to smaller uncertainties in the position of the interference fringes, whereas an increase of the shear distance can lead to an exposure of the interferometer to larger phase differences. Due to the nonlinear character of the SIR method, it is difficult to predict a priori how and to what extent the reconstructions can benefit from such changes. For now, it can only be stated that the relative change in the shear distance and the visibility was too small to show a perceivable impact, especially in comparison with potential effects from systematic uncertainties like beam fluctuations.

**Figure 9:**
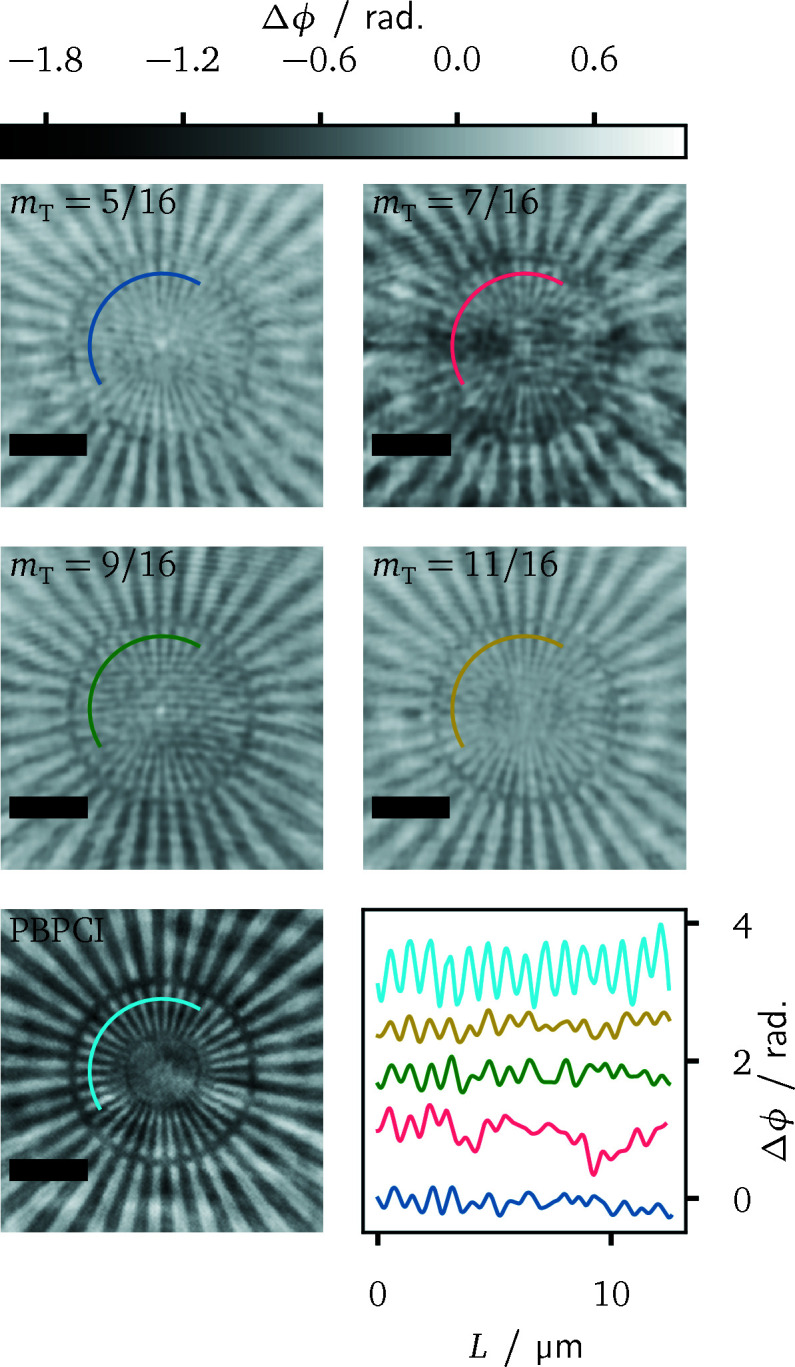
Phase reconstructions of a Siemens star for different Talbot orders and for PBPCI. The scale bar indicates 5 µm length in all reconstructions. The two visible indicators within the Siemens star mark spoke line widths of 500 nm and 200 nm, respectively. The panel on the bottom right shows section profiles along the indicated circular line segments. The length *L* of the line segments, that the phase shift values are plotted over, is measured anti-clockwise. In support of a clearer depiction, the section profiles are offset with respect to each other.

While grating interferometry could achieve a slightly better CNR, the visually best reconstruction in Fig. 8 ▸ is obtained from propagation-based phase contrast. Most notably, the latter yields a higher spatial resolution, as can be inferred most clearly from the smaller disks and the thin line segments of the DESY-logo. A more quantitative assessment of the spatial resolution between the different methods will be presented now. It is based on the reconstructions of a Siemens star, which is also composed of a 500 nm gold layer on the same membrane as the DESY test pattern. It comprises 36 spokes with line sizes from 2 µm in the outer region down to 50 nm in the centre. Indicators mark line sizes of 500 nm, 200 nm and 100 nm. Fig. 9 ▸ illustrates central regions of the reconstructions as well as section profiles along a circular path through each reconstruction. Two aspects stand out in particular. First, the superior spatial resolution of propagation-based phase contrast imaging is confirmed, as the spokes of the Siemens star can be resolved further towards the centre and up to the indicator of 200 nm line width. The latter is hardly visible itself in the grating-based reconstructions. Secondly, the achieved resolution is direction-dependent. Finer spoke widths can be resolved in the horizontal direction. This anisotropy is more pronounced for grating interferometry. In comparison, there is only a miniscule degradation for PBPCI.

**Figure 10:**
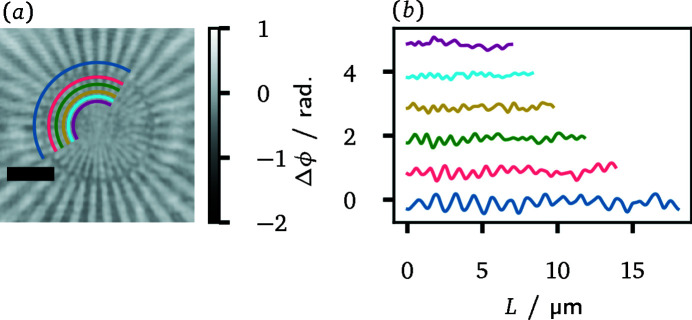
Illustration for the determination of the resolution estimates Δ*r*_h_ and Δ*r*_v_ at the example of grating interferometry in the Talbot order *m*_T_ = 11/16. The scale bar in the reconstruction (*a*) indicates 5 µm length. Panel (*b*) shows multiple section profiles along the circular segments marked in (*a*). The section profiles are offset with respect to each other and the length *L* of the line segments, that the phase shift values are plotted over, is measured anti-clockwise.

In order to find more accurate resolution estimates Δ*r*_h_ and Δ*r*_v_ for the horizontal and the vertical direction, multiple circular section profiles with different radii were analyzed. This procedure is exemplified in Fig. 10 ▸ for the case *m*_T_ = 11/16. The circular segments cover an angle of 150° or 15 periods of the Siemens star. As the resolution estimate in the vertical direction Δ*r*_v_, the half-period of the section with the largest radius is chosen for which the expected number of periods cannot be unambiguously determined. Note that the proper identification of the periods always fails in the vertically oriented part of the line segment. In the presented example, this is the case for the yellow profile in Fig. 10 ▸(*b*) with an expected half-period of 330 nm. The resolution estimate Δ*r*_h_ for the horizontal direction, on the other hand, is set to the half-period over the segment with the smallest radius that still exhibits a clearly periodic modulation. As in the light blue profile of the example with a half-period of 280 nm, a clearer modulation always occurs at the beginning of the section profile corresponding to the horizontally oriented part of the circular segment. The resolution estimates obtained for the other configurations are listed in Table 2 ▸. If no single section profile could be identified for a resolution estimate, the range between the half-periods from two adjacent segments is given.

An alternative resolution estimate for propagation-based imaging was obtained using the Fourier ring correlation criterion (Van Heel & Schatz, 2005 ▸). Here, two independent reconstructions have to be correlated. They were obtained by splitting up the ten frames of each exposure series into two parts. The crossover of the 1/2-bit threshold then indicates a resolvable half-period of 210 nm. This is consistent with the estimates obtained from the visual analysis of the Siemens star. For the results with the SIR method, the same approach was not applicable. Here, the correlation remained above the threshold over the entire spatial frequency range, *i.e.* up to half-periods corresponding to the effective pixel size of 53 nm. It is assumed that the correlation at higher spatial frequencies is induced by the regularization, even if they do not carry any information about the sample.

A possible explanation of the anisotropy observed for the propagation-based method is the effective numerical aperture of the focusing element. Since approximately half of the FZP is covered by the beamstop, the numerical aperture in the vertical direction is also halved. A resolution limit follows from the requirement that diffracted X-rays corresponding to a spatial frequency ν should still be able to interfere with the non-diffracted beam, leading to λ*z*_SD_ν ≤ 2*z*_FD_NA with NA the effective numerical aperture. For the setup at hand, this implies resolvable half-periods of 75 nm in the horizontal and of 150 nm in the vertical direction. While neither of the two limits is achieved in practice, they still can explain the observed direction-dependence. Regarding the grating-based setup, there are two further aspects that can account for the larger anisotropy of the spatial resolution as well as for the slight decrease of Δ*r*_v_ with higher Talbot orders. First, an anisotropic version of the TV norm, *cf*. equation (8), was employed in the SIR method. It penalized differences in the vertical direction ten times as much as those along the horizontal. Secondly, as mentioned in the *Introduction* [Sec sec1], the extraction of Fourier orders from a single interferogram is only well defined if they are band-limited such that higher resolutions can be obtained for smaller fringe periods. While the SIR method is not based on the extraction of Fourier orders, it might still be able to recover higher spatial frequency information regarding the sample when the fringe period is smaller. Nevertheless, it should be emphasized that the resolution obtained in the vertical direction still outperforms the limitation of the Fourier transform method. The half-period resolvable with the latter is limited to Δ*r*_FT_ = *p*_f_/*M*_SD_, *i.e.* the demagnified fringe period. Overall, the SIR method does not completely resolve this stringent limitation to the resolution of single-exposure grating-based interferometry, but it manages to mitigate it considerably.

## 5. Summary and outlook

In summary, single-exposure X-ray phase imaging microscopy with a grating interferometer was demonstrated in an experiment using synchrotron radiation. Previous implementations of grating-based X-ray microscopy were based on X-ray imaging microscopes, where the sample is placed in a conjugate plane upstream of the X-ray optics, and relied on the phase stepping approach and hence on the acquisition of multiple frames (Yashiro *et al.*, 2009 ▸, 2010 ▸). In comparison, we could demonstrate a broader applicability of grating interferometry through a statistical image reconstruction method. On the one hand, this allowed the adaption of the method in X-ray projection microscopes, where the sample is placed in the divergent X-ray beam and the phase retrieval is applied to the Fresnel diffraction image of the sample. On the other hand, our algorithmic approach also mitigated limitations to the spatial resolution when applying grating interferometry in a single-exposure scheme.

We have analyzed the image quality in terms of quantitativeness and the contrast-to-noise ratio of the reconstructed phase images as well as in view of the achieved spatial resolution, each on the basis of test samples with known compositions, namely carbon fibres and nanostructured resolution test patterns. This evaluation was carried out for different parameters of the grating interferometer. With the exception of a single exposure, namely the case *m*_T_ = 9/16 in Fig. 8 ▸, a quantitative phase contrast manifests in all reconstructions. For the contrast-to-noise ratio, a clear dependence on neither the shear distance of the interferometer nor the visibility of the interference pattern could be revealed. We suspect that this is largely due to systematic errors, for instance due to variations of the X-ray wavefront between sample and reference measurements. Regarding this question, further experiments, covering a larger domain of the parameter space and ideally featuring fewer systematic variations, are necessary. Finally, it can be also suspected here that the choice of the regularization parameters within our reconstruction method partly determines the achievable contrast-to-noise ratio, and that adaptions of the regularization are required in order to transfer improvements of the signal-to-noise ratio from the data to the reconstructions. The spatial resolution as inferred from reconstructions of a Siemens star still exhibits deteriorations for larger fringe periods. In comparison with reconstruction techniques based on the Fourier transform method (Takeda *et al.*, 1982 ▸), however, the observed dependency is substantially less pronounced. The comparative imaging experiments with propagation-based phase contrast achieved a superior spatial resolution at a comparable contrast-to-noise ratio.

Besides the inconsistency of the illumination, the predominant errors in the reconstructions from grating interferometry were constituted by stripe artefacts caused by non-uniformities of the interference fringe pattern. Hereof, it can be expected that our method will benefit from future improvements regarding the manufacturing of micro- and nanometric X-ray gratings resulting in transmission functions that fit the assumption of perfect periodicity more properly. Regarding future applications of grating interferometry in imaging microscopes, it was demonstrated that the usage of an order sorting aperture can become the limiting factor regarding the spatial resolution of the microscope. In such cases, it seems favourable to dispose of the order sorting aperture and to tolerate potentially intrusive contributions of higher diffraction orders of the Fresnel zone plate instead. Alternatively, other focusing elements such as compound refractive lenses (Schroer *et al.*, 2005 ▸; Seiboth *et al.*, 2018 ▸) could be used. Another limiting factor for grating interferometry can be the orientation of sample features with respect to the grating bars, *cf*. Fig. 7 ▸ and the discussion at the end of Section 4.2. A potential solution to this issue is offered by two-dimensionally structured X-ray gratings which then offer a bidirectional sensitivity. In the field of X-ray microscopy, for instance, this method was already employed by Berujon *et al.* (2012 ▸). In general, the benefits of bidirectional sensitivity should become most obvious for use cases where the sample does not fully fit into the field of view. Then, unidirectional methods might miss phase information that bidirectional ones can recover. The price to pay for bidirectional sensitivity, at least in single-exposure applications, is a limitation of the spatial resolution by the period length of the grating in both directions. As we could demonstrate a mitigation of such limitations for the unidirectional case with our statistical image reconstruction method, its combination with two-dimensionally structured gratings seems promising. A straightforward extension, however, can be impeded by increasing computational costs, as the number of terms to compute in equation (5) grows quadratically with the number of considered grating diffraction orders (Wolf *et al.*, 2020 ▸). Finally, further extensions of the technique might also benefit from more suited regularization schemes. As the transfer function in the phase difference regime exhibits zeroes for integer multiples of the spatial frequency 1/*d*_s_ (Takano *et al.*, 2019 ▸; Wolf *et al.*, 2020 ▸), a first step can be the specific incorporation of these frequencies into the regularization term rather than the general demand of smoothness used thus far.

Inconsistencies during the reference correction caused by beam or pulse variations play an even more important role for experiments at X-ray free-electron lasers. There, intrinsic pulse-to-pulse fluctuations occur due to the stochastic nature of the self-amplified spontaneous emission (SASE) process. Associated changes of the intensity or the pointing of the beam then impede the recording of proper data for the reference correction. For propagation-based phase contrast imaging, Hagemann *et al.* (2021 ▸) approached this problem by generating synthetic reference frames from a principal component analysis over a pre-recorded series of single-pulse empty beam images. In this context, grating interferometry has the advantage that the phase retrieval step solely operates on a single exposure, whereas both sample and empty beam data are required in the propagation-based method. While reference information is still required for the isolation of the sample transmission function, grating interferometry offers the possibility of extending the idea of synthesized reference data to the space of complex wavefields. First tests and characterizations regarding the application of synthesized reference frames to X-ray grating interferometry should be the subject of future experiments. In case of a successful adaption, X-ray grating interferometry can be a promising alternative for full-field imaging within the challenging environment posed by fluctuating SASE pulses.

## Figures and Tables

**Table 1:** Overview of the distances *z*_FD_ and 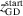 obtained from the fits to the inverse grating magnification values

*m*_T_	*z*_FD_ (m)	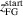 (m)
5/16	14.454 ± 0.010	0.4558 ± 0.0005
7/16	14.432 ± 0.018	0.5606 ± 0.0009
9/16	14.44 ± 0.06	1.030 ± 0.004
11/16	14.43 ± 0.06	1.240 ± 0.005

**Table 2:** Summary of different setup and reconstruction quality parameters for the realized Talbot orders *m*_T_: the shear distance *d*_s_ of the grating interferometer, the fringe period *p*_f_, the visibility *V* ^(2)^ corresponding to terms with *n* − *m* = 2 from equation (5), the contrast-to-noise ratio (CNR), the resolution estimates Δ*r*_h_ and Δ*r*_v_ in the horizontal and the vertical direction, and the resolution limit Δ*r*_FT_ of the Fourier transform method in the vertical direction due to the fringe period

*m*_T_	*d*_s_ (µm)	*p*_f_ (µm)	*V* ^(2)^	CNR	Δ*r*_h_ (nm)	Δ*r*_v_ (nm)	Δ*r*_FT_ (µm)
5/16	172	134	0.41	7.92	280–330	400	1.10
7/16	170	98	0.39	3.00	280–330	390–460	0.78
9/16	166	70	0.28	5.74	280–330	330	0.57
11/16	164	59	0.31	8.06	280	330	0.48
PBPCI	–	–	–	7.47	200–230	230	–
